# Sustainable Development and Storage Stability of Orange By-Products Extract Using Natural Deep Eutectic Solvents

**DOI:** 10.3390/foods11162457

**Published:** 2022-08-15

**Authors:** Clara Gómez-Urios, Adriana Viñas-Ospino, Pablo Puchades-Colera, Daniel López-Malo, Ana Frígola, María José Esteve, Jesús Blesa

**Affiliations:** 1 Nutrition and Food Science, Faculty of Pharmacy, University of Valencia, Avenida Vicent Andrés Estellés, s/n, 46100 Burjassot, Spain; 2 Faculty of Health Science, European University of Valencia, 46100 Valencia, Spain

**Keywords:** natural deep eutectic solvents, green extraction, orange peels, bioactive compounds, polyphenols, COSMOTherm

## Abstract

The citrus industry produces large amounts of waste rich in bioactive compounds that have important effects on human health. Their extraction was performed using organic solvents, and a greener alternative to those solvents are natural deep eutectic solvents (NADES). The present study aimed to obtain and optimize extracts rich in polyphenols and flavonoids from orange peels using NADES and monitor polyphenol stability in the extracts for 30 days. The software COSMOtherm (conductor-like screening model) was used to screen fourteen NADES. The most promising solvents were lactic acid:glucose (LA:Glu) with an extraction yield of 1932 ± 7.83 mgGAE/100 gdw for TPC (total polyphenol content) and 82.7 ± 3.0 mg/100 gdw for TFC (total flavonoid content) and in the case of L-proline:malic acid (LP:MA) was 2164 ± 5.17 mgGAE/100 gdw for TPC and 97.0 ± 1.65 mg/100 gdw for TFC. The extraction process using LA:Glu and LP:MA was optimized, and the results showed that the selected variables (%NADES, solid:liquid ratio, and extraction time) had a significant influence on the extraction of TPC and TFC. Results showed that NADES improve the stability of TPC. These findings revealed that NADES are efficient for the extraction of bioactive compounds from orange by-products, and these extracts can represent an alternative for the food industry to enrich food products with natural ingredients.

## 1. Introduction

Global orange production is estimated at around 115.5 million tons, and from this amount, Spain produces over 3.3 million tons [1]. Fifty percent of the orange weight is by-products, and these are discarded, representing an important environmental problem. Orange by-products include peel (flavedo and albedo), pulp, and seeds, which are known sources of valuable compounds with biological effects on human health [2,3]. For example, orange peel extracts have been demonstrated to decrease the risk of developing cancer in mouse models [4]. These biological benefits can be attributed to the wide range of biomolecules present in orange peels as fermentable sugars, carbohydrate polymers, flavonoids, polyphenols, vitamins, carotenoids, and essential oils [5]. Especially, polyphenols have gained much interest due to their antioxidant and inflammatory effects [6,7].

Polyphenol extraction has been developed in the industry using a different kind of organic solvents. However, these conventional extractions require a large amount of solvent, which causes problems such as environmental pollution and toxic effects on human health [8,9]. For this reason, the implementation of new techniques using green solvents to replace petrochemical solvents is a concern. According to one of the principles of green chemistry, reducing energy consumption by energy recovery and using innovative technologies with non-pollutant solvents is a priority [10]. A green extraction process must be free from hazardous organic solvents or use a reduced quantity of organic solvents. In recent years, natural deep eutectic solvents (NADES) have gained interest as an alternative to replace organic solvents [11]. NADES are a mix of components that exist in nature and act as hydrogen bond acceptors (HBA) or hydrogen bond donors (HBD) [12]. Additionally, NADES have several advantages: they are non-flammable, miscible with water, easily degradable, biocompatible, non-toxic, and have high extraction power for different polar substances in plants [13]. However, the main problem with NADES is their viscosity, which limits the extraction power. To reduce the viscosity, water can be added in different amounts, and this variable has been well-studied to optimize the extraction conditions in different matrices [11,14,15]. 

For the extraction process of bioactive compounds, the selection of the optimal solvent considering their physicochemical affinity is a crucial step. In the case of citrus fruits, polar phenolic chemicals are more prevalent than nonpolar ones. There exist two categories to classify phenolic substances: glycosylated flavones and polyethoxylated flavones. Hesperidin is often the flavonoid that is most abundant in the group of citrus fruits including in the skins [7,16]. However, the process of searching a suitable solvent for a specific compound is time- and energy-consuming. To solve this problem, there exists software than can evaluate the solubility of a molecule in a wide range of solvents. COSMOtherm software has been used before to screen different solvents, including NADES, for the extraction of catechin [17] and carotenoids [8]. The results have demonstrated that this software can predict the solubility of the before-mentioned molecules very closely. 

Nowadays, consumers are more concerned about including in their diet more natural products with biological benefits in health [6]. Considering this trend, the industry has a focus on searching for new ways to formulate food products enriched with natural-based bioactive compounds. At this point is where NADES fits and can help the food industry to solve this challenge. One of the most important advantages of almost all NADES is that their components are considered generally recognized as safe (GRAS) by the Food and Drug Administration (FDA) [18], and the extracts obtained (NADES-bioactive compound) can be included directly in food products and enhance the nutritional value of the final product. NADES and also the pure components have been demonstrated to have antioxidant properties, biological activity, and antiproliferative effects [15,19,20]. For example, choline chloride, a usual component of NADES, has demonstrated a positive association between its consumption and the risk of developing a different kind of cancer [21]. Furthermore, the components of NADES are present in nature and are present in our daily diet (e.g., choline, citric acid, betaine, amino acids, and sugars) [22].

Based on the information discussed above, the aim of the present study is to screen fourteen NADES using the software COSMOtherm and select the most promising solvent for polyphenol and flavonoid extraction from orange peels. Additionally, to obtain extracts rich in bioactive and develop an efficient extraction process, the selected NADES were optimized, and TPC stability was monitored during storage. The extracts obtained are based on NADES with safe compounds, and it can be expected that their inclusion in food product formulation can enrich nutritional, bioactive value and improve shelf life.

## 2. Materials and Methods

### 2.1. Raw Material

Peels were obtained from orange fruits (*Citrus sinensis*, Navel cultivar) donated by a local agricultural cooperative (Carlet, Spain). The oranges were washed with distilled water and used immediately. The orange peels were removed from the pulp and milled with a kitchen grinder (Lacor-60344). In parallel, orange peel was dried in an oven at 100 ± 0.2 °C to constant weight to study the dry weight.

### 2.2. Chemical and Reagents

The chemicals used in the study were obtained from the suppliers described below: choline chloride (ChChl) (≥98%), D-(-)-fructose (Fruc) (≥99%), α-D-glucose (Glu) (96%), D-(+)-xylose (Xy) (≥99.0%), DL-lactic acid (LA) (90%), Red Nile, and Folin–Ciocalteau reagent were purchased from Sigma-Aldrich (Steinheim, Germany). Glycerol (Gly) was from Glentham Life Science (Corsham, UK). DL-malic acid (MA) (≥98%) was from Thermo Fisher (Kendel, Germany). L-(+)-tartaric acid (TA) (>99.0%) was purchased from TCI (Zwijndrecht, Belgium). Citric acid (CA) (≥99.9%), anhydrous sodium carbonate (Na_2_CO_3_), and sodium nitrite (NaNO_2_) were purchased from VWR Chemicals (Leuven, Belgium). L-proline (LP) was from Guinama (Valencia, Spain). Betaine (Bet) was acquired from Fluorochem (Hadfield, UK), and aluminum chloride (AlCl_3_.6H_2_O) was purchased from Acofarma (Terrassa, Barcelona). Absolute ethanol was purchased from J.T Baker Chemical Co. (Deventer, The Netherlands).

### 2.3. COSMOtherm Simulation

Software BIOVIA COSMOtherm 2020 version 20.0.0 (Dassault Systems, Paris, France) was used for the solubility calculations of hesperidin in NADES. Software BIOVIA TmoleX19 version 2021 (Dassault System, Paris, France) was used for the geometric and energetic optimization of NADES compounds and hesperidin used in the present study.

COSMOtherm software was used to simulate the solubilization of hesperidin in different NADES. The first step was the optimization of the geometry and density of molecules using discrete Fourier transform (DFT). For this purpose, each molecule was optimized using the COSMO-BP-TZVP template of the Tmolex software package (interface of TUR-BOMOLE). Then, COSMO calculations were performed using the software BIOVIA COSMOtherm 2020 version 20.0.0 with BP_TZVP_C30_19.CTD parametrization. COSMOtherm software was used as a tool to predict the activity coefficient of hesperidin in 100%, 85%, 75%, 50%, and 30% of NADES at 60 °C.

### 2.4. Preparation of Natural Deep Eutectic Solvents

Natural deep eutectic solvents were prepared according to the method of Dai et al. (2015) [11] with some modifications. NADES were prepared by mixing the reagents in specific molar ratios and then stirred at 80 °C in a water bath until a transparent liquid was formed. Fourteen different NADES systems with two components were obtained, and different amounts of distilled water were added and stirred to reduce viscosity and facilitate the migration of the bioactive compounds from the matrix to the solvent. NADES were kept in darkness in sealed glass flasks at room temperature until use. Table 1 shows the composition, molar ratios, and acronyms of the NADES used in this study.

### 2.5. NADES Characterization

pH measure was made with pH meter Sension + MM340 (Hach, Germany). The polarity of NADES was measured by Red Nile (1 g/L ethanol) (Steinheim, Germany), adding 50 µL of the colorant to the cuvette and measuring its absorbance in the interval from 400 to 700 nm (spectrophotometer, Perkin Elmer^®^, Boston, MA, USA). Maximum absorption wavelength (λ_max_) was extrapolated in Equation (1). The results were expressed in kcal mol^−^^1^.

(1) $$E_{NR}=\frac{hcN_{A}}{\lambda_{max}}$$

where, *h* represents Planck’s constant (6.63 × 10^−34^ J·s), *c* speed of light in a vacuum (2.997 × 10^8^ m/seg) and *N_A_* Avogadro’s constant (6.02214076 × 10^23^ mol^−1^).

### 2.6. Extraction Procedure

For model validation, orange peel samples were placed in a beaker with 75% NADES (*v*/*v*) in a ratio of 1:10 for 30 min. The extraction was done by magnetic stirring and heating; the temperature was 45 ± 5 °C for each sample. The samples were then centrifuged in a 5810 R centrifuge (Eppendorf, Germany) at 5 °C, 3000 rpm for 30 min. The supernatant layer was stored in dark tubes at 4 °C until analysis. All the extractions were performed in triplicate.

### 2.7. Total Polyphenol Content by UV–Vis Spectroscopy

Total polyphenol content (TPC) of orange peel extracts was carried out using the method described by Singleton and Rossi (1965) [23]. An aliquot of the sample (100 µL) was mixed with 3 mL of sodium carbonate (2%, *w*/*v*) and 100 µL of Folin–Ciocalteu reagent (1:1, *v*/*v*). Gallic acid calibration curves were done under the same conditions as samples. After 1 h of reaction at room temperature, the absorbance was measured at a wavelength of 765 nm (spectrophotometer, Perkin Elmer **^®^**, Boston, MA, USA). The content of total polyphenols was made in mg of gallic acid equivalent (GAE) per 100 g of DW.

### 2.8. Total Flavonoid Content by UV–Vis Spectroscopy

The total flavonoid content (TFC) of orange peel was determined using the method of Zhishen (1999) [24]. An aliquot of 100µL of the sample was mixed with 1088 µL of ethanol (30%, *v*/*v*) and 48 µL of sodium nitrite (0.5 mol/L) and vortexed. After 5 min of reaction, 48 µL of aluminum chloride (0.3 mL/L) was added. The sample was able to react for 5 min, and 320 µL of sodium hydroxide (1 mL/L) was added and vortexed again. The absorbance was measured at a wavelength of 510 nm spectrophotometer, Perkin Elmer **^®^**, Boston, MA, USA). Catechins (2 mg/mL) calibration curve was carried out under the same conditions as the samples. TFC results were expressed in mg of catechin equivalents (CE) per 100 g of DW.

### 2.9. Optimization Process

After the model validation, two NADES (LA:Glu and LP:MA) were selected for the optimization process. The parameters of optimization for TPC and TFC from orange peels were performed by an RSM. A five-level and three factors, Box–Behnken design consisting of twenty experimental runs was employed including four replicates at the center point. The extraction variables were solid:liquid ratio (X_1_, 5–20 mL), %NADES (X_2_, 30-85), and extraction time (X_3_, 5–30 min). The data were analyzed using the Design Expert program (11.0 version), and the coefficients were interpreted using *F*-test, and a quadratic model was used to build response surfaces. The adequacy of the model was determined by evaluating the lack of fit, coefficient of determination (R^2^), and the Fisher test value (*F*-value) obtained from the analysis of variance (ANOVA). Finally, the significate interaction between the variables studied was represented in 3D plots.

### 2.10. TPC Stability

The effect of storage time and temperature on TPC in NADES extracts and ethanol as control were monitored. Extracts were stored at 25 °C and 4 °C. The extracts were analyzed for total phenolic content for 30 days. The degradation rate was calculated (C/C_0_), where C_0_ is the initial phenolic concentration, and C is the phenolic concentration after storage.

### 2.11. Statistical Analysis

All measures and experiments were repeated three times. Response surface plots were generated with Design-Expert 8.0 for Windows**^®^** (Stat-Ease, Minneapolis, MN, USA). The analysis of variance (ANOVA) and the post hoc test Tukey were performed using the software SPSS Statistics, version 26.0.0 (SPSS Inc., Chicago, IL, USA). Differences at *p* < 0.05 were considered significant.

## 3. Results

### 3.1. NADES Characterization

The extraction solvent should be selected considering parameters such as safety, price, availability, and affinity with the target compound. Table 1 shows the characteristics of each NADES at 75% mixed with water (75:25, *v*/*v*) and the molar ratios used. Is well-studied that NADES physicochemical properties influence the relationship with the target bioactive compounds as reported by [25].

The different pH values of NADES may affect the hydrogen bond interactions among solute and NADES. Most of the fourteen NADES studied have acidic pH; moreover, the acidity of each NADES is due to its components. Those solvents made with acidic components (carboxylic group) have lower pH values compared with the ones made with sugar (carbonyl groups), which have the highest pH values. NADES can donate and accept hydrogen bonds and electron pairs, giving the capacity to make hydrogen bonds between NADES and solvents [26].

Polarity and pH are physicochemical characteristics that affect the extraction of bioactive compounds and their solubilizing capabilities, where a high ENR value indicates a low polarity, and acidic pH can be advantageous to extract polar compounds [27]. For example, Dai et al. [28] found that LP:MA mixed with water (75:25, *v*/*v*) resulted in optimum for the extraction of TPC from *Carthamus tinctorius* L. due to its polarity, which is similar to methanol and ethanol.

### 3.2. COSMOtherm Simulation and Model Validation

To design a green and efficient extraction process, the first step is the selection of the most suitable solvent for a target compound, and the second is the optimization of the extraction method [25]. In this way, the process of selection of an optimal solution can be time- and energy-consuming; in consequence, it can increase the costs. For this reason, COSMOtherm software was employed to screen 14 NADES for the extraction of TPC and TFC from orange peels. Hesperidin was selected as the target compound because it is the most abundant flavonoid present in orange peels [16,29]. Consequently, to evaluate the accuracy of the software, the model was experimentally validated with the suitable and unsuitable NADES for the extraction of hesperidin.

**Figure 1:**
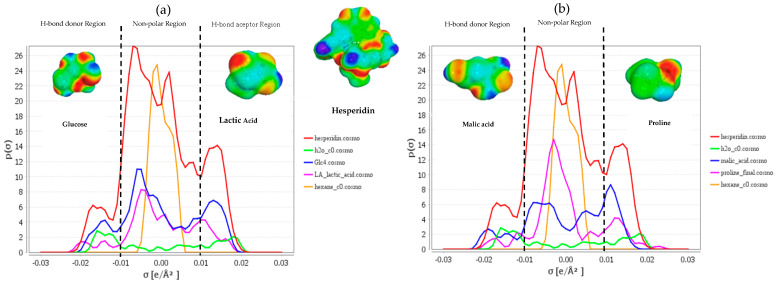
(**a**) Sigma profile of hesperidin (red), water (green), glucose (blue), lactic acid (pink), and hexane (orange). (**b**) Sigma profile of hesperidin (red), water (green), malic acid (blue), L-proline (pink), and hexane (orange) and their sigma surfaces. H-bond, hydrogen bond.

One of the outputs that COSMOtherm can create is the sigma profile, and this can help to understand better the polarities and affinity of a molecule for NADES compounds. Figure 1 represents the sigma profile for hesperidin in LA:Glu (Figure 1a) and LP:MA (Figure 1b). The profiles are divided into three quadrants with corresponding σ values: the HBD, the nonpolar region, and the HBA region. Negative values represent positive polarities and vice versa. The peaks around 0 e/A^2^ show the apolarity of hesperidin, and the peaks from 0.01 to 0.03 and −0.01 to −0.03 show that this polyphenol also includes polar regions. It can be observed that the best NADES to dissolve polyphenols are polar ones. Additionally, when hexane is represented in the sigma profile, the peak is in the non-polar region, reflecting the low affinity for this polyphenol.

For the screening of NADES using the COSMOtherm, first, it was necessary to simulate the solubility of hesperidin with the 14 NADES in the software’s option-activity coefficient (lnγ) calculation. The calculation of the activity coefficient was performed using 100%, 85%, 75%, 50%, 40%, and 30% of NADES in water (*v*/*v*). Table 2 shows the results of NADES screening: when there is a low activity coefficient of the tested NADES, there exists a higher hesperidin solubility (green color); in contrast, when the values of activity coefficient are high, there exists a poor hesperidin solubility (orange color). It can be observed that there are higher values of lnγ in NADES formed with acid compounds (LA:Glu, LP:MA, and MA:Glu), and for their acidic nature, their pH is also very low. In addition, the predictions showed lower lnγ in NADES based on choline chloride, reflecting a low solubility of hesperidin and also corresponding with the higher pH values. In Table 2, it is demonstrated how the solubility of hesperidin can change with different amounts of NADES in water, where the %NADES is proportional to the solubility of hesperidin. The COSMOtherm predictions were in good agreement with other studies, where acid-based NADES had better extraction efficiency than polyphenols [20,30]. For example, Radosevic et al. [30] reported better extraction of polyphenols from grape skins using acid-based NADES. Another physicochemical property that should be taken into account is polarity, as organic acid-based NADES are more polar than sugar and polyalcohol-based ones. In consequence, NADES with high polarity showed better efficiency with polar compounds such as polyphenols, anthocyanins, and flavonoids [22,25,31,32].

**Figure 2:**
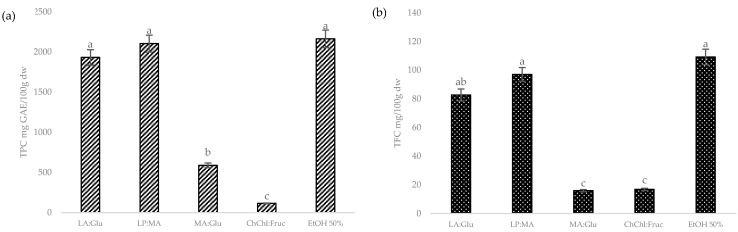
Model validation. (**a**) Total polyphenol content (mg GAE/100 gdw), (**b**) total flavonoid content (mg/100 gdw). Results are expressed as the means (*n* = 3) ± DS. Different lowercase letters (a–c) are significantly different (*p* < 0.05) as measured by Tukey’s test.

To demonstrate the accuracy of COSMOtherm software and validate the model, extracts were prepared with the NADES with the highest and lowest lnγ values. Extractions were prepared with 75% of NADES because the addition of water has been demonstrated to decrease the viscosity of NADES and, in consequence, enhance the mass transfer from plant matrices to the solution, increasing the extraction efficiency [22]. The selected NADES for the validation were lactic acid:glucose (LA:Glu), L-proline:MA (LP:MA), malic acid:glucose (MA:Glu), and choline chloride:fructose (ChChl:Fruc). Furthermore, to compare the results, an extraction with EtOH (50% *v*/*v*) was performed as a control. Total polyphenols (TPC) and total flavonoids (TFC) were measured to validate the model, and the results are shown in Figure 2 and were in good concordance with the prediction of COSMOtherm. There was no significative difference (*p* < 0.05) between LA:Glu (1932 ± 7.83 mgGAE/100 gdw and 82.7 ± 3.01 mg/100 gdw), LP:MA (2164 ± 5.17 mgGAE/100 gdw and 97.0 ± 1.65 mg/100 gdw), and EtOH extract (2164 ± 8.97 mgGAE/100 gdw and 109 ± 5.22 mg/100 gdw) for TPC and TFC, respectively. These results highlight the possibility of NADES replacing conventional solvents such as ethanol for polyphenol and flavonoid extraction. In contrast, the lowest extraction efficiencies were in ChChl:Fruc for TPC and in ChChl:Fru and MA:Glu for TFC. This was also expected due to the fact that ChChl:Fruc has sugar and belongs to the group of solvents with pH values close to 6, which does not favor the extraction of polar compounds. Based on these results, it is possible to conclude that activity coefficient calculation in COSMOtherm software is a good tool to predict the solubility of hesperidin in NADES.

Based on the results, efficient extraction yields and physicochemical affinity for the target compound were important conditions for solvent selection. However, another aspect should be considered before the next optimization process. The availability and safety are important for their potential inclusion in the industry. The compounds of NADES are safe, and LA, Glu, LP, and MA are part of our diet and have positive effects in health. Lactic acid, proline, glucose, and malic acid are considered GRAS and are already present in food products. Additionally, these NADES compounds have biological activity by themselves; for example, lactic acid is present in yoghurt, cheese, and kefir and can improve gut health. The FDA has approved its use in most products apart from infant foods and formula. In the case of malic acid, there is no scientific basis for suspecting that the amounts of malic acid added to foods would be toxic according to the Select Committee on GRAS Substances (SCOGS) [33]. Proline is an amino acid and has been also related with positive biological effects as anti-tumor, anti-fungal, and antimalarial [34].

### 3.3. Optimization of Extraction Conditions by Response Surface Methodology

The optimization process is the second step for an efficient extraction, and for this reason, the most promising NADES selected in the model validation (LA:Glu and LP:MA) were optimized considering TPC and TFC as response variables. The RSM was performed for optimization of the extraction parameters using Design Expert 11.0. Solvent-to-solid ratio (5 to 25 mL), %NADES (85% to 30% (*v*/*v*)), and extraction time (5 to 25 min) were the variables considered in the optimization due to their high impact in TPC and TFC; coded levels and independent variables are shown in Table 3. The %NADES (*v*/*v*) have a direct impact in the extraction efficiency and, due to the addition of water, can help to reduce the viscosity of NADES and improve the mass transfer to the solvent [11].

To evaluate the effect of the considered variables in the responses, a Box–Behnken design was used. The experimental values using the presented model considering the two selected NADES for each compound are shown in Supplementary Materials Table S1.

ANOVA was used to calculate the significance of the quadratic model of all independent variables and is summarized for LA:Glu in Supplementary Materials Table S2 and for LP:MA in Supplementary Materials Table S3. The determination coefficient (R^2^) 0.99 indicates that models provide credible fit values for the extraction of TPC and TFC from orange peels using LA:Glu and LP:MA. Additionally, lack of fit in the RSM model of the responses were greater than 0.05 (non-significant) in all the cases, which means that the model fits well, and there is significant effect on output responses. In addition, the coefficients of variation were between 1.78 and 5.85, indicating that the results were reliable and accurate. Then, the values of the coefficients for TPC and TFC for each NADES were used for a final predictive equation. The regression equations were highly significant (*p* < 0.05), and the final polynomials equations in terms of actual factors were:

#### 3.3.1. Lactic Acid: Glucose

*TPC* = 352.54 × *X*_1_ + 609.34 × *X*_2_ + 338.59 × *X*_3_ − 283.80 × *X*_1_*X*_2_ + 241.24 × *X*_2_*X*_3_ − 545 × *X*_1_^2^ 399.44 × *X*_2_^2^ + 579.71 × *X*_3_^2^ (2)

*TFC* = 127.77 *−* 15.01 × *X*_1_ − 58.79 × *X*_2_ + 68.80 × *X*_3_ 14.12 × *X*_1_*X*_2_ + 9.01 × *X*_2_*X*_3_ + 15.89 × *X*_1_^2^ + 18.62 × *X*_2_^2^ − 34.19 × *X*_3_^2^ (3)

#### 3.3.2. L-Proline: Malic Acid

*TPC* = 3828.25 *+* 189.02 × *X*_1_ − 1895.33 × *X*_2_ + 1425.09 × *X*_3_ + 241.16 × *X*_1_*X*_2_ −     718.06 × *X*_1_*X*_3_ − 1019.80 × *X*_2_*X*_3_ − 1610.97 × *X*_1_^2^ + 739.78 × *X*_2_^2^ + 908.10 × *X*_3_^2^ (4)

*TFC* = 137.52 − 44.77 × *X*_1_ − 66.97 × *X*_2_ + 80.30 × *X*_3_ + 8.77 × *X*_1_*X*_2_ − 38.94 × *X*_1_*X*_3_
    − 34.59 × *X*_2_*X*_3_ + 9.51 × *X*_1_^2^ + 12.80 × *X*_2_^2^ − 20.81 × *X*_3_^2^ (5)

where *TPC* is the total polyphenolic content, *TFC* is total flavonoid content, *X*_1_ is the solid/liquid ratio, *X*_2_ is %NADES, and *X*_3_ is extraction time.

According to the *p*-values, which were <5 for all variables, they had a statistically significant influence on TPC and TFC, and this demonstrated the significance of the presented models.

**Figure 3:**
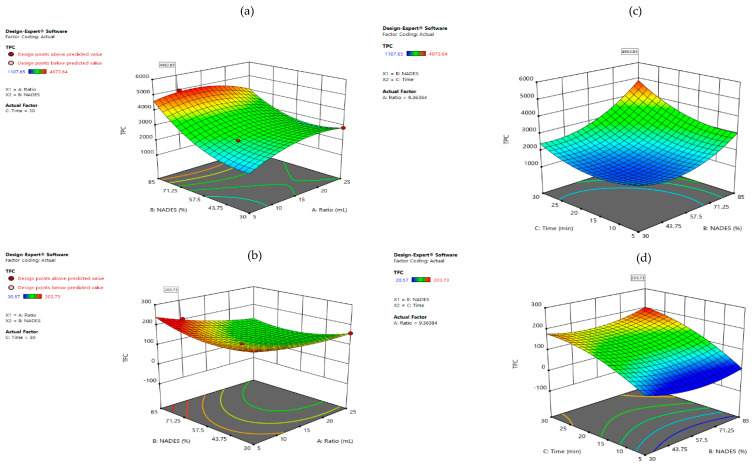
Response surface plots showing combined significative effects on TPC (**a**,**b**) and TFC (**c**,**d**) using LA:Glu as solvent.

Response surface plots were created to show just the significative interactions between two variables on the response values. In Figure 3 are shown the 3D plots using LA:Glu as solvent for TPC and TFC. In Figure 2a are presented the response surfaces for TPC generated first by the %NADES and solid/liquid, and it was observed that by increasing the % of NADES and decreasing the ratio, a higher yield of TPC is achieved.

This can be explained because when higher % of NADES exist, the hydrogen bond interaction between the target compound and solvent are stronger, and the addition of water in more than 50% may be considered an aqueous solution, and the components can be dissociated [17]. In the second interaction for TPC, it is clear that longer extraction time and high %NADES can increase the extraction yields. A short extraction time can be insufficient for some bioactive compounds, but a longer extraction time can produce degradation of the extracted compounds [35]. In other studies, it has been reported that extraction yield can increase with longer extraction time but also can decrease after long periods [36]. Moreover, long periods of time extraction can contribute to the degradation of bio compounds, and a high solvent-to-solid ratio can increase the dispersion of the solvent in the sample, reducing the extraction performance [37,38].

**Figure 4:**
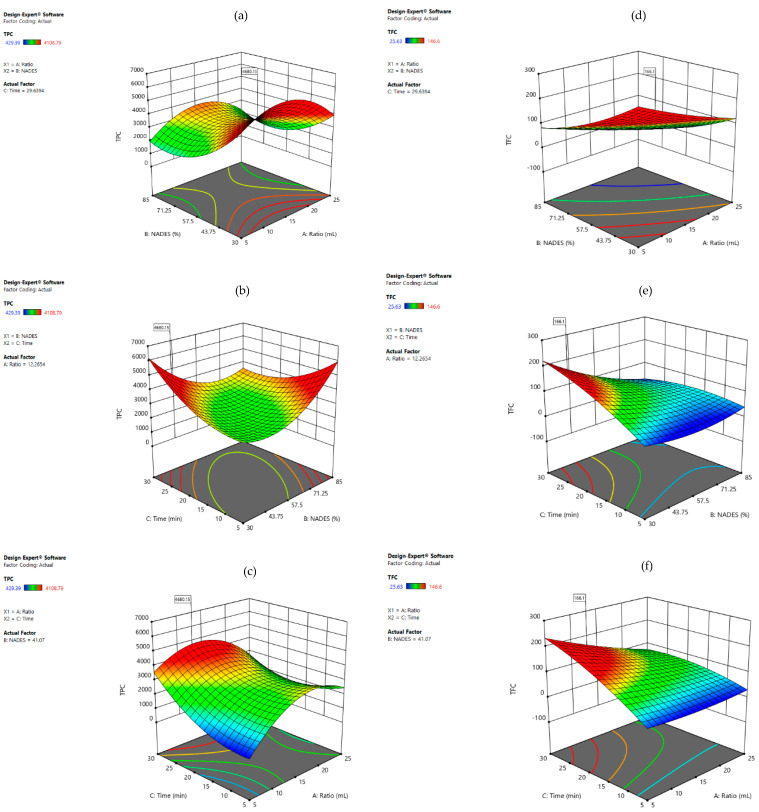
Response surface plots showing combined effects of solid:liquid ratio, %NADES, and extraction time for TPC (**a**–**c**) and TFC (**d**–**f**) with LP:MA as solvent.

In Figure 4b are presented three response surfaces for TFC, and as was expected, they showed the same tendency as TPC, where higher %NADES and solid:liquid ratio increased the extraction yields, and the interaction between %NADES and extraction time was directly proportional. In Figure 4 are shown the 3D plots using LP:MA as solvent for the optimization of TPC and TFC, and in this case, all the interactions between the variables were significative (*p* < 0.05). First, Figure 4a shows the response surfaces for TPC, where the tendency was different when compared with LA:Glu. A lower %NADES showed better extraction yields, and when the extraction time increased, %NADES also became higher. This can be explained due to the high viscosity of LP:MA, which can reduce the extraction efficiency, and when the extraction time is longer, the viscosity can be reduced [15].

**Figure 5:**
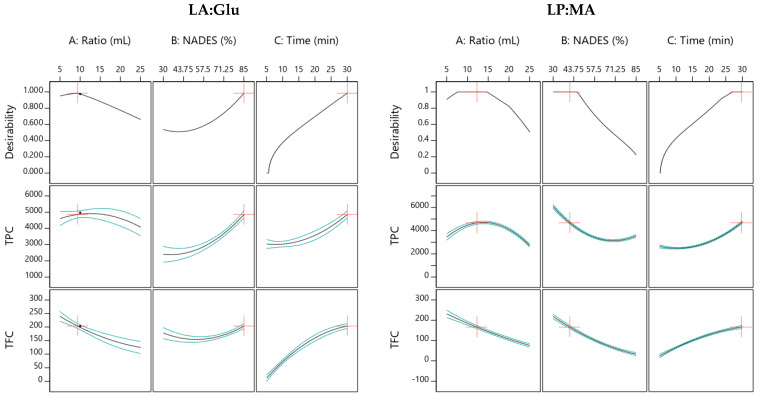
Optimal conditions for the extraction of TPC and TFC simultaneously using LA:Glu and LP:MA as solvent. A: ratio (mL per 1 g of peel); B: NADES in water (%, *v*/*v*); C: time extraction (min).

The final goal of RSM was the process optimization where the developed models can be used to predict the TPC and TFC. By applying the desirability function and considering the maximum responses, the optimal conditions for the extraction of TPC and TFC simultaneously from orange peels were calculated using LA:Glu and LP:MA. In the first case, the optimal conditions are represented in Figure 5 and were 9.36 mL of solid:liquid ratio, 85% of NADES, and 30 min of extraction time. Under these optimal conditions, the extraction yield of TPC was 4862 mg GAE/100 gdw, and in this case, this NADES extracted a higher concentration of TPC than in other similar studies [39] and for TFC 203 mg/100 gdw. For the second optimization using LP:MA, results are presented in Figure 5, where the optimal conditions were a solid:liquid ratio of 12.2 mL, %NADES of 41.1, and extraction time of 29.6 min; the extraction yields in these conditions were 4680 mg GAE/100 gdw for TPC and 166.1 mg/100 gdw for TFC. Considering these results, it can be clearly observed that optimal parameters can change in different solvents, and it is associated with their physicochemical properties. LP:MA has higher viscosity (0.0150 Pas) [19]; for this reason, it is expected that higher % of NADES in water can reduce the extraction yields.

In addition, in both solvent the extraction, the time was around 30 min, which is in accordance to other studies that suggest that optimum time for the extraction of polyphenolic compounds should be between 30 to 180 min, but it is dependent on the characteristics of the samples [7].

However, extraction time is an important parameter for the industrial application, and due to prolonged operation, they are difficult to be applied on a large scale and could lead to polyphenols oxidation. Prabowo et al. [40] found that extraction time affected the extraction yield of TPC. Finally, the solvent:solid ratio was slightly low, which can be explained by the mass transfer principles. It can be concluded with the results presented above that the considered conditions for the optimization in the present study had a strong effect in the extraction of TPC and TPFC. The validation of the suggested extraction process and the optimal conditions were tested using the same methodology as optimization samples and was confirmed with a deviation of ±5.

### 3.4. TPC Stability

**Figure 6:**
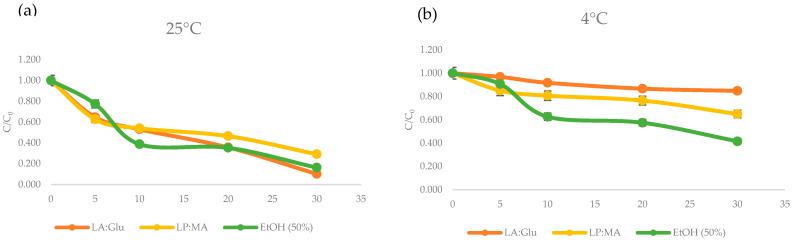
Stability of phenols in NADES (La:Glu, LP:MA) and ethanol (50% *v*/*v*) extracts at 25 °C (**a**) and 4 °C (**b**) during storage for 30 days. Results were expressed as the means (n = 3) ± SD.

One important aspect in the selection of the most appropriate solvent is the stability of the extracted compound in the solvent, and this parameter is an important aspect if the final application includes these extracts in food formulation. The stability was monitored in LP:MA and La:Glu and also with the extraction performed using ethanol (50% *v*/*v*). In Figure 6 are presented the results for TPC during storage for 30 days in different conditions: at 25 °C and 4 °C. The results demonstrated decreased values of TPC in all NADES extracts at room temperature, where TPC was more stable in LP:MA extracts, with 70% of degradation. Then, at low temperatures, TPC in LA:Glu showed degradation of 15% at 4 °C and LP:MA 35% after 30 days. In all the storage conditions, DES extracts showed better stability of polyphenols when compared with ethanol extract. Other studies have reported that DES contributes to the stabilization of phenolic compounds. Panic et al. [19] reported that extracts with choline chloride:citric acid enhances the stability of 70% of anthocyanins at 4 °C. Additionally, Dai et al. [41] reported that LA:Glu systems provide better stability of cyanidin when compared to ethanol extractions. These results can be explained due to the interaction between the phenolic compounds and the components of NADES mixtures, which decrease the movement of solutes molecules and reduce their contact time with oxygen, and in consequence, the oxidative degradation is reduced [41,42]. The enhanced stability of TPC in NADES extracts indicates their potential application in the fortification of food products, and this characteristic can contribute to extending the shelf life of the enriched products.

## 4. Conclusions

In this study, green extracts rich in bioactive compounds (polyphenols and flavonoids) from orange peel were obtained following the principles of green chemistry using sustainable solvents. The screening of 14 NADES using COSMOtherm software was performed to select the most suitable solvent for the extraction of TPC and TFC from orange peel. The experimental validation corroborated that the software is a good tool to predict the solubilization of bioactive compounds in NADES. LA:Glu and LP:MA showed the most promising results for TPC and TFC extraction, and the process was optimized considering solid:liquid ratio, %NADES, and extraction time as variables. The optimization results showed that the considered variables have a strong effect on the extraction of TPC and TFC, but they can change depending on the solvent. Additionally, NADES demonstrated to enhance polyphenol stability during 30 days of storage. In this study, the NADES studied might be considered a good alternative to replace other organic solvents for the extraction of bioactive compounds from orange peel. In addition, the proposed extracts have the potential to be included in industrialized food products due to their natural components and safe consumption. Future research should be directed at the study of the physicochemical characterization of final food products with the inclusion of NADES extracts.

## Figures and Tables

**Table 1:** Used NADES and their physicochemical characteristics.

Acronym	Hydrogen Bond Acceptor	Hydrogen Bond Donor	Molar Ratio	pH	Polarity (kcal/mol)
ChChl: Glu	Choline chloride	Glucose	2:1	4.14 ± 0.02	49.79 ± 0.27
ChChl: Fruc	Choline chloride	Fructose	1.9:1	4.35 ± 0.01	51.26 ± 0.30
ChChl: Xy	Choline chloride	Xylose	2:1	4.26 ± 0.01	49.21 ± 0.10
ChChl: Gly	Choline chloride	Glycerol	1:2	5.19 ± 0.00	49.00 ± 0.17
ChChl: MA	Choline chloride	Malic acid	1:1	1.62 ± 0.03	47.85 ± 0.05
ChChl: TA	Choline chloride	Tartaric acid	2:1	1.54 ± 0.02	47.98 ± 0.01
ChChl: LA	Choline chloride	Lactic acid	1:3	1.43 ± 0.02	47.97 ± 0.01
ChChl: CA	Choline chloride	Citric acid	2:1	1.45 ± 0.02	47.81 ± 0.00
ChChl: LP: MA	Choline chloride	L-Proline: Malic acid	1:1:1	2.11 ± 0.00	48.87 ± 0.01
LA: Glu	Lactic acid	Glucose	5:1	1.16 ± 0.04	47.89 ± 0.04
MA: Glu	Malic acid	Glucose	1:1	1.34 ± 0.02	47.57 ± 0.00
LP: MA	L-Proline	Malic acid	1:1	2.22 ± 0.01	48.30 ± 0.03
Bet: CA	Betaine	Citric acid	1:1	2.36 ± 0.00	47.97 ± 0.02
Bet: MA	Betaine	Malic acid	1:1	2.08 ± 0.01	48.79 ± 0.02

Polarity and pH were expressed as the means (n = 3) ± SD.

**Table 2:** Predicted ln γ_solutes_ for hesperidin in 70% NADES (*v*/*v*) using COSMOtherm.

Acronym	ln(γ) 85% NADES	ln(γ) 75% NADES	ln(γ) 50% NADES	ln(γ) 40% NADES	ln(γ) 30% NADES
EtOH	−1.063				
LA: Glu	−4.52	−4.02	−3.66	−3.31	−2.24
LP: MA	−4.73	−3.78	−3.28	−2.83	−1.71
MA: Glu	−4.81	−3.67	−2.94	−2.31	−0.93
ChChl: LA	−2.15	−1.88	−1.60	−1.29	−0.28
Bet: MA	−3.60	−2.12	−1.20	−0.55	0.47
ChChl: LP: MA	−1.44	−1.11	−0.75	−0.30	0.61
ChChl: Xyl	−2.49	−1.73	−0.87	0.00	1.78
Bet: CA	−0.53	−0.26	−0.12	0.06	0.70
ChChl: MA	−1.16	−0.60	−0.07	0.44	1.31
ChChl: TA	−2.14	−1.10	−0.33	0.48	1.74
ChChl: Glu	−2.24	−1.35	−0.34	0.53	2.04
ChChl:CA	−1.52	−0.66	0.02	0.75	1.93
ChChl: Gly	−0.60	−0.21	0.28	0.77	1.62
ChChl: Fruc	−1.01	−0.51	0.11	0.82	1.89

Gray color, reference; green, better or equivalent solvent than reference; yellow color, slightly worse solvent than reference; red color, worse solvent than reference. ChChl, choline chloride; Glu, glucose; Fruc, fructose; Xyl, xylose; Gly, glycerol; MA, malic acid; TA, tartaric acid; LA, lactic acid; CA, citric acid; LP, L-proline; Bet, betaine.

**Table 3:** Coded levels and independent variables.

Independent Variable			Level			
		−∞	−1	0	+1	+∞
Solid:liquid ratio	X_1_	5	10	15	20	25
NADES (%, *v*/*v*)	X_2_	30	40	50	75	85
Extraction time	X_3_	5	10	15	20	30

## Data Availability

Data is contained within the article or supplementary material.

## Supplementary Materials

The following supporting information can be downloaded at: https://www.mdpi.com/article/10.3390/foods11162457/s1, Table S1: Box-Behnken design with the independent variables and responses data of the optimization for the suitable NADES for each bioactive compound; Table S2: Analysis of variance (ANOVA) for the fitted quadratic polynomial model for optimization of TPC and TFC using LA: Glu as solvent; Table S3: Analysis of variance (ANOVA) for the fitted quadratic polynomial model for optimization of TPC and TFC using LP:MA as solvent of squares.
