# Supplementary material for: Sustainable Development and Storage Stability of Orange By-Products Extract Using Natural Deep Eutectic Solvents
Source: Foods. 2022 Aug 15;11(16):2457. doi: 10.3390/foods11162457 (PMC9407522; doi:10.3390/foods11162457)
Supplement: Supplementary file 1 [file foods-11-02457-s001.zip › foods-1837709-supplementary.pdf]

Table S1. Box-Behnken design with the independent variables and responses data of the optimization for the suitable NADES for each bioactive compound.

Run	Ratio (mL) X_1	NADES (%) X_2	Time (min) X_3	LA:Glu		LP:MA	
				TPC (mgGAE/100g$_{dw}$)	TFC (mg/100g$_{dw}$)	TPC (mgGAE/100g$_{dw}$)	TFC (mg/100g$_{dw}$)
1	15	50	10	1520	31.1	2804	46.8
2	25	75	15	1740	38.8	2824	42.7
3	5	50	5	1398	23.7	429	28.5
4	15	30	15	1925	113.3	3582	120.2
5	5	85	5	2956	46.0	3381	33.0
6	25	30	20	1942	28.9	2569	41.8
7	10	50	30	2687	153.2	3712	146.6
8	15	30	15	1920	109.2	3580	120.5
9	15	30	15	1921	116.3	3578	121.3
10	10	75	30	3873	167.3	2990	70.0
11	20	30	5	1753	29.9	2645	39.4
12	25	85	15	2001	52.4	3895	51.1
13	10	85	30	4973	203.7	3232	45.3
14	5	75	5	2124	22.7	1954	25.6
15	5	40	20	ND	ND	ND	ND
16	25	30	30	2852	159.7	4021	123.1
17	25	50	20	1107	56.5	1561	50.9
18	15	75	10	2360	20.6	4108	41.2
19	15	30	15	1922	112.6	3577	124.0
20	5	30	5	1435	47.4	786	47.45

X_1: solid/liquid ratio, X_2: %NADES, X_3: extraction time, TPC: Total phenolic content, TFC: Total flavonoid content, ND: Not detected, Glu: glucose, MA: malic acid, LA: lactic acid, LP: L-proline.

Table S2: Analysis of variance (ANOVA) for the fitted quadratic polynomial model for optimization of TPC and TFC using LA: Glu as solvent.

LA:Glu						
	TPC			TFC		
**Source**	**SS**	**F-value**	***p*-value**	**SS**	**F-value**	***p*-value**
**Model**	1533.71	133.91	< 0.0001*	61492.02	306.60	< 0.0001*
X_1-Ratio	1741.50	13.69	0.0049*	315.53	14.16	0.0045*
X_2-NADES	3723.52	29.28	0.0004*	3465.88	155.53	< 0.0001*
X_3-Time	2037.53	16.02	0.0031*	8410.14	377.40	< 0.0001*
X_1X_2	4626.54	36.38	0.0002*	1144.33	51.35	< 0.0001**
X_1X_3	37855.13	2.98	0.1186^{ns}	18.56	0.8330	0.3852^{ns}
X_2X_3	2253.52	17.71	0.0023*	314.33	14.11	0.0045*
X_1^2	2577.57	20.27	0.0015*	218.35	9.80	0.0121*
X_2^2	1317.60	103.60	< 0.0001*	2862.31	128.44	< 0.0001*
X_3^2	2650.51	20.84	0.0014*	921.90	41.37	0.0001*
**Residual**	1145.54			200.56		
Lack of Fit	96524.88	4.31	0.0911^{ns}	174.50	5.36	0.0645^{ns}
Pure Error	17930.91			26.06		
**C.V. %**	5.05			5.85		
**R^2**	0.99			0.99		
**Adjusted R^2**	0.98			0.99		

TPC: Total phenolic content, TFC: Total flavonoid content, Glu: glucose, LA: lactic acid. Significant at $p<0.05$, ns Not significant at $p>0.05$. C.V: Coefficient of variation, SS: Sum of squares.

Table S3: Analysis of variance (ANOVA) for the fitted quadratic polynomial model for optimization of TPC and TFC using LP:MA as solvent of squares.

	LP:MA					
	TPC			TFC		
**Source**	**SS**	**F-value**	***p*-value**	**SS**	**F-value**	***p*-value**
**Model**	1999.70	826.03	< 0.0001*	29938.42	293.83	< 0.0001*
X_1-Ratio	50035.89	18.61	0.0020*	2807.40	247.98	< 0.0001*
X_2-NADES	36020.60	1339.90	< 0.0001*	4497.46	397.26	< 0.0001*
X_3-Time	3609.60	1342.27	< 0.0001*	11458.21	1012.11	< 0.0001*
X_1X_2	3340.50	124.24	< 0.0001*	441.28	38.98	0.0002*
X_1X_3	7233.50	269.03	< 0.0001*	2127.45	187.92	< 0.0001*
X_2X_3	4025.60	1497.23	< 0.0001*	4631.05	409.06	< 0.0001*
X_1^2	2245.60	834.95	< 0.0001*	78.23	6.91	0.0274^{ns}
X_2^2	4519.60	1680.77	< 0.0001*	1352.80	119.49	< 0.0001*
X_3^2	6505.50	241.95	< 0.0001*	341.76	30.19	0.0004*
**Residual**	24197.37			101.89		
Lack of Fit	21241.88	5.75	0.0575^{ns}	90.07	6.10	0.0522^{ns}
Pure Error	2955.49			11.82		
**C.V. %**	1.78			4.84		
**R^2**	0.99			0.99		
**Adjusted R^2**	0.99			0.99		

TPC: Total phenolic content, TFC: Total flavonoid content, LP: L-proline**,** MA: malic acid. Significant at p<0.05, ns Not significant at p>0.05. C.V: Coefficient of variation, SS: Sum.
